# The European System for Cardiac Operative Risk Evaluation (EuroSCORE) is not appropriate for withholding surgery in high-risk patients with aortic stenosis: a retrospective cohort study

**DOI:** 10.1186/1749-8090-4-32

**Published:** 2009-07-14

**Authors:** Dimitri Kalavrouziotis, Debbie Li, Karen J Buth, Jean-Francois Légaré

**Affiliations:** 1 Department of Surgery, Division of Cardiac Surgery, Queen Elizabeth II Health Sciences Centre, Dalhousie University, 1796 Summer Street, room 2269, Halifax, Nova Scotia, B3H 3A7, Canada

## Abstract

**Background:**

The European System for Cardiac Operative Risk Evaluation (EuroSCORE) is a widely used risk assessment tool in patients with severe aortic stenosis to determine operability and to select patients for alternative therapies such as transcatheter aortic valve implantation. The objective of this study was to determine the accuracy of the EuroSCORE in predicting mortality following aortic valve replacement (AVR).

**Methods:**

The logistic EuroSCORE was determined for all consecutive patients that underwent conventional AVR between 1995 and 2005 at our institution. Provincial Vital Statistics were used to determine all-cause mortality. The accuracy of the prognostic risk prediction provided by logistic EuroSCORE was assessed by comparing observed and expected operative mortality.

**Results:**

During the study period, a total of 1,421 patients underwent AVR including 237 patients (16.7%) that had a logistic EuroSCORE > 20. Among these patients, the mean predicted operative mortality was 38.7% (SD = 18.1). The actual mortality of these patients was significantly lower than that predicted by EuroSCORE (11.4% vs. 38.7%, observed/expected ratio 0.29, 95% CI 0.15–0.52, P < 0.05). The EuroSCORE overestimated mortality within all strata of predicted risk. Although medium-term mortality is significantly higher among patients with EuroSCORE > 20 (log rank P = 0.0001), approximately 60% are alive at five years.

**Conclusion:**

Actual operative mortality in patients undergoing AVR is significantly lower than that predicted by the logistic EuroSCORE. Additionally, medium-term survival following AVR is acceptable in high-risk patients with EuroSCORE > 20. More accurate risk prediction models are needed for risk-stratifying patients with severe aortic stenosis.

## Introduction

Aortic valve replacement (AVR) is established therapy for the treatment of severe, symptomatic aortic stenosis (AS) and has been shown to relieve symptoms and significantly improve survival [1-4]. Conventional AVR involves median sternotomy and the institution of cardiopulmonary bypass, excision of the stenotic native valve, and replacement with a biologic or mechanical prosthesis. Recently, catheter-based transfemoral and transapical approaches to aortic valve implantation have been developed for patients with severe AS that are thought to be inoperable because of advanced age or significant comorbidities, with acceptable early results [5-13].

In these studies, patient selection for transcatheter therapy was determined largely on the basis of the European System for Cardiac Operative Risk Evaluation (EuroSCORE). The EuroSCORE is a surgical risk scoring system developed in 1999 from a multinational European database [14]. The model provides an estimate of a patient's anticipated 30-day mortality according to the patient's demographic characteristics, cardiovascular and non-cardiovasculr risk factors, and procedural variables. Its predictive accuracy has previously been validated in a variety of clinical settings [15-17]. However, concerns have been raised about the EuroSCORE's ability to accurately predict outcomes of patients at the extremes of risk and patients undergoing valve surgery [18-22].

In order to assess the accuracy of the prognostic risk prediction provided by EuroSCORE, we examined the operative and long-term mortality of a contemporary, real-world cohort of patients with a pre-procedural logistic EuroSCORE > 20 (which represents a predicted operative mortality risk of 20%) that have undergone conventional AVR.

## Methods

### Study design and patients

A retrospective cohort design was used. All consecutive adult patients who underwent conventional AVR between March 1995 and September 2005 at our institution were identified. Indications for AVR were based on clinical symptoms and disease severity and were consistent with current consensus guidelines [4]. The selection of patients for AVR is the result of a weekly round-table peer-review process involving cardiologists, cardiac surgeons, and cardiac radiologists, during which patients with severe symptomatic AS are discussed and the timing of operation established according to a previously published algorithm [23].

Baseline patient demographics, cardiovascular and non-cardiovascular comorbid illnesses, as well as data on intra-operative variables and postoperative in-hospital events were captured in a prospective fashion in our institutional database. The database is audited annually, has < 5% missing data fields, and is free of systematic error [24]. Definitions of variables and outcome measures in the database are consistent with the standards published by the Society of Thoracic Surgeons [25].

The logistic EuroSCORE was computed for each patient in the study using the β coefficients of the logistic regression equation reported for the EuroSCORE [14,26]. The score thus obtained corresponds exactly to the operative mortality predicted for a given patient, defined as death occurring anytime during the same postoperative hospital stay of any duration or within 30 days of surgery for patients discharged to home or to a secondary institution. The distribution of demographic, clinical, and operative variables was compared among patients with EuroSCORE ≤ 20 and those with EuroSCORE > 20. A EuroSCORE cut-off of 20, representing a predicted operative mortality of 20%, was chosen a priori to define low- and high-risk patient subgroups on the basis of previously published reports evaluating the safety, feasibility, and efficacy of transcatheter aortic valve implantation in which patients with EuroSCORE > 20 were considered to have too high a risk to be candidates for conventional AVR and were subsequently referred for catheter-based aortic valve implantation [5-13,27].

### Clinical outcomes

The primary outcome of interest in this study was operative mortality, defined as death within 30 days of operation or within the same hospital admission. Because our institutional database captures in-hospital mortality only, we linked survivors to discharge to government vital statistics data to determine 30-day, 1, 3, and 5-year survival. Survival times were analyzed using Kaplan-Meier methodology and log-rank statistics were used to compare survival curves among patients with logistic EuroSCORE > 20 and those with logistic EuroSCORE ≤ 20 [28].

Other postoperative adverse events examined were: dependence on a mechanical ventilator for a period exceeding 24 hours, stroke, the transfusion of banked red blood cells, new-onset atrial fibrillation, the implantation of a permanent pacemaker secondary to new, postoperative complete heart block, and length of hospitalization from the time of operation to discharge. Stroke was defined as a permanent neurologic deficit occurring after surgery with an acute change on intracranial computed tomography or magnetic resonance imaging.

Postoperative delirium and acute, reversible deficits were not included in the stroke end-point. Individual outcomes were univariately compared among patients with logistic EuroSCORE ≤ 20 and those with logistic EuroSCORE > 20. The accuracy of the prognostic risk prediction provided by EuroSCORE was assessed by comparing observed and expected operative mortality. Ninety-five percent confidence intervals (CI) for the observed-to-expected ratios (O/E) were generated using Byar's approximation [29].

### Statistical analysis

Statistical analysis for the study was performed using Statistical Analysis System (SAS) software version 8.2 (SAS Institute, Cary, North Carolina). Continuous variables are expressed as mean ± 1 standard deviation (SD). Continuous variables were compared using unpaired t-tests or non-parametric equivalents where appropriate. Proportions were compared using chi-square tests (or Fisher's exact test where appropriate) and Mantel-Haenszel chi-square statistics with one degree of freedom were used to test for trends across ordered categories. Comparisons were considered statistically significant if the P value was less than 0.05. All P values were two-sided.

The study protocol was in accordance with the institutional clinical research ethics board and received full ethical approval.

## Results

### Baseline characteristics

Between 1995 and 2005, 1,421 patients underwent AVR at our institution. Among these patients, 237 (16.7%) had a logistic EuroSCORE > 20. Patients with logistic EuroSCORE > 20 were more likely to have a number of high-risk features such as advanced age, depressed systolic function of the left ventricle, and chronic renal insufficiency, although were not different from patients with logistic EuroSCORE ≤ 20 with respect to gender, or the prevalence of diabetes mellitus, hypertension, hyperlipidemia, and active or former cigarette smoking (Table 1). In addition, the acuity of operation, the type of prosthesis implanted, the need for concomitant revascularization or thoracic aortic repair, and operative durations also differed among the two groups (Table 2).

**Table 1:** Baseline characteristics of patients with EuroSCORE > 20 compared to those with EuroSCORE ≤ 20

**Variable, % unless otherwise indicated**	**EuroSCORE > 20,** **n = 237**	**EuroSCORE ≤ 20,** **n = 1,184**
Demographic data		
Age		
<60 yrs	11.4	24.7
60–69 yrs	13.9	25.4
70–79 yrs	42.6	37.3
≥ 80 yrs	32	12.6
Age, mean yrs ± SD	73.2 ± 12.4	66.7 ± 12.8
Female	35.0	33.8
Cardiovascular risk factors/diseases		
Current or former cigarette smoking	57.0	61.5
Body mass index, mean kg/m^2 ^± SD	26.6 ± 4.6	28.2 ± 5.3
Active endocarditis	8.0	1.1
Diabetes mellitus	22.8	21.5
Hypertension	59.9	53.3
Extracardiac arteriopathy	56.5	18.6
Pulmonary hypertension	31.2	5.2
Previous cardiac surgery	30.4	7.9
Left ventricular ejection fraction		
>0.50	37.6	80.4
0.30–0.50	35.4	16.4
<0.30	27.0	3.2
Unstable angina	14.4	0.8
MI within 21 days of surgery	13.1	2.6
Hyperlipidemia	48.5	49.1
Medical comorbidities		
Hemoglobin, mean g/dL ± SD	12.2 ± 1.9	13.5 ± 1.7
Serum creatinine > 200 μmol/L	15.2	1.9
Chronic lung disease	33.3	15.0

MI = myocardial infarction; SD = standard deviation.

**Table 2:** Operative characteristics of patients with EuroSCORE > 20 compared to those with EuroSCORE ≤ 20

**Variable, % unless otherwise indicated**	**EuroSCORE > 20,** **n = 237**	**EuroSCORE ≤ 20,** **n = 1,184**
Critical preoperative state*	24.5	1.0
Emergency surgery	10.1	0.9
Concomitant thoracic aortic surgery	16.5	5.7
Concomitant CABG	58.7	42.2
Type of prosthesis		
Bioprosthesis (tissue valve) Mechanical valve Homograft	84.0 13.5 2.5	81.7 17.6 0.8
Cardiopulmonary bypass time, mean min ± SD	178.5 ± 64.4	148.8 ± 51.8
Aortic clamp time, mean min ± SD	122.8 ± 47.0	106.7 ± 37.8
Prosthetic valve indexed EOA§<0.75 cm^2^/m^2^	4.7	10.4

*Includes: resuscitated cardiac arrest, dependence on mechanical ventilator, and cardiogenic shock requiring inotropic support and/or intra-aortic balloon counterpulsation.

§Effective orifice area was computed for each implanted valve type and size according to the measurements published by valve manufacturers and/or independent adjudicators and was indexed on patient body surface area.

CABG = coronary artery bypass graft surgery; EOA = effective orifice area; SD = standard deviation.

### Early outcomes

The overall operative mortality, defined as death occurring anytime during the same postoperative hospital stay or within 30 days of surgery for patients discharged to home or to a secondary institution, for the entire cohort of patients was 4.6% (n = 66). The operative mortality was significantly higher among patients with logistic EuroSCORE > 20 compared to those with EuroSCORE ≤ 20 (11.4% vs. 3.2%, respectively, P < 0.0001). Of the pre-specified in-hospital adverse events following surgery examined, only the incidence of new-onset postoperative atrial fibrillation was similar between the two groups (Table 3).

**Table 3:** Early postoperative outcomes of patients with EuroSCORE > 20 compared to those with EuroSCORE ≤ 20

**Outcome, %**	**EuroSCORE > 20,** **n = 237**	**EuroSCORE ≤ 20,** **n = 1,184**	**P**
Mortality (in-hospital or within 30 days of surgery)	11.4	3.2	<0.0001
In-hospital events			
Ventilation > 24 hours	27.9	9.3	<0.0001
Length of stay > 9 days	59.1	28.1	<0.0001
Stroke	5.1	2.3	0.02
Atrial fibrillation (new onset)	31.7	28.4	0.31
Erythrocyte transfusion	73.0	34.5	<0.0001
Permanent pacemaker implantation	10.1	5.4	0.01

**Figure 1:**
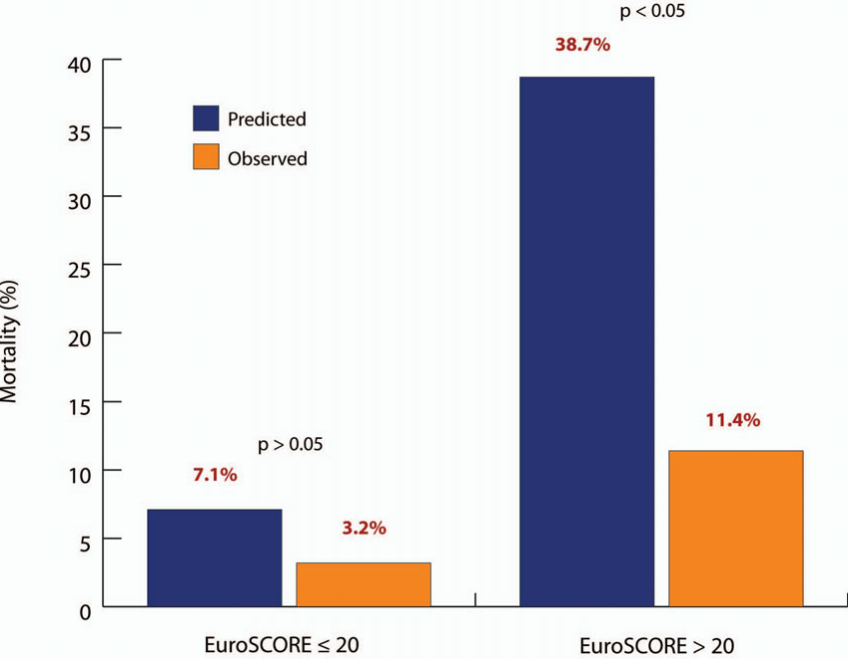
**Predicted and observed mean operative mortality among patients with EuroSCORE > 20 compared to those with EuroSCORE ≤ 20**. Among high-risk patients with logistic EuroSCORE > 20, the actual operative mortality following AVR was significantly lower than that predicted by EuroSCORE (11.4% vs. 38.7%, respectively, O/E 0.29, 95% CI 0.15–0.52, P < 0.05).

**Figure 2:**
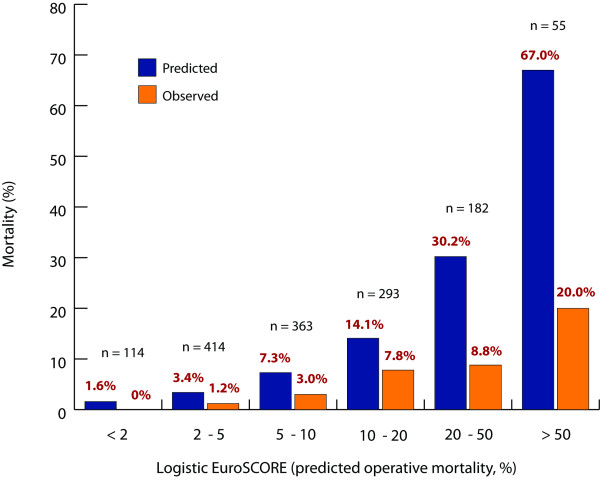
**Predicted and observed mean operative mortality within all subgroups of EuroSCORE**. The EuroSCORE overestimated operative mortality in all categories of risk.

Among the subgroup of patients with logistic EuroSCORE > 20, the mean predicted operative mortality by EuroSCORE was 38.7% ± 18.1%. The actual mortality of these patients was significantly lower than that predicted by EuroSCORE (11.4% vs. 38.7%, O/E 0.29, 95% CI 0.15 – 0.52, P < 0.05) (Figure 1). In the lower-risk subgroup of patients with logistic EuroSCORE ≤ 20, mean predicted operative mortality was 7.1% ± 4.8%. The observed mortality in these patients was 3.2%, although this difference was not statistically significant (O/E 0.47, 95% CI 0.10 – 1.30, P > 0.05). In addition, the EuroSCORE overestimated operative mortality within all strata of predicted risk, the magnitude of the discrepancy getting larger with increasing risk (Figure 2).

**Figure 3:**
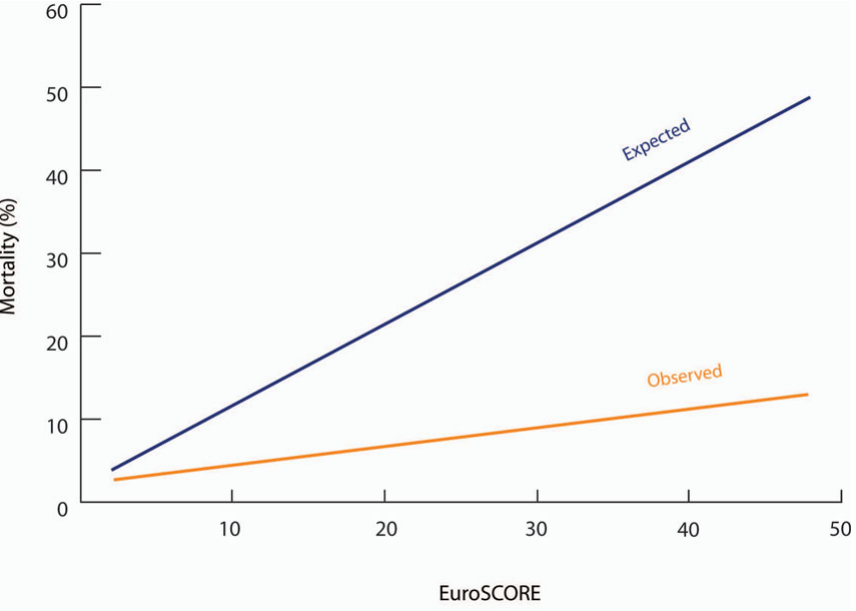
**Simple linear regression model of observed mortality as a function of logistic EuroSCORE compared with predicted mortality**. There is a statistically significant association between logistic EuroSCORE and actual operative mortality (r^2 ^= 0.95), although the empiric model is different from the theoretical model provided by EuroSCORE.

A simple linear regression model of actual mortality was fit as a function of EuroSCORE as the independent variable and compared to the predicted mortality given by EuroSCORE (Figure 3). Although there is a statistically significant association between EuroSCORE and operative mortality (r^2 ^= 0.95) such that increasing EuroSCORE is associated with increasing mortality, the two curves are widely divergent and the difference between them increases with increasing predicted risk.

### Late outcomes

**Figure 4:**
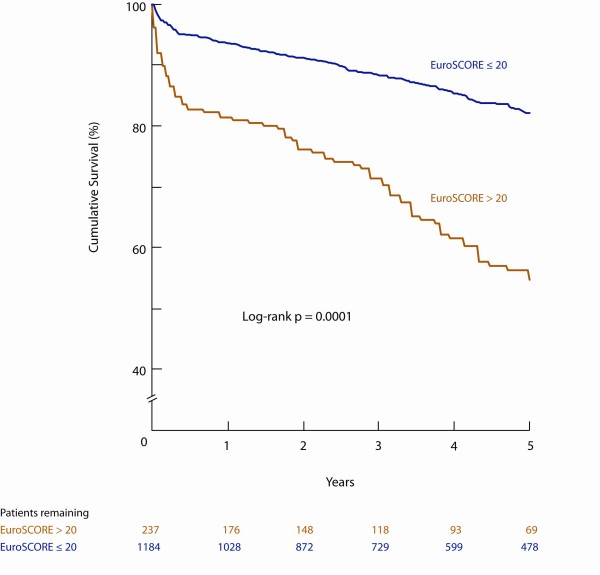
**Five-year freedom from all-cause mortality**. The Kaplan-Meier survival among patients with logistic EuroSCORE > 20 is significantly worse compared to those with logistic EuroSCORE ≤ 20.

Patients were linked to vital statistics databases to determine mortality from any cause. The Kaplan-Meier freedom from all-cause mortality was significantly lower for patients with logistic EuroSCORE > 20 compared to those with logistic EuroSCORE ≤ 20 at 1, 3, and 5 years: 80.9% vs. 93.5%, 68.5% vs. 87.9%, and 56.2% vs. 82.1%, respectively (log-rank P = 0.0001) (Figure 4).

## Discussion

The purpose of this study was to determine the early and medium-term outcomes of a contemporary cohort of patients with severe symptomatic AS undergoing AVR and to evaluate the accuracy of the EuroSCORE in predicting operative mortality. We have shown that in a tertiary care surgical practice in Canada a significant proportion of patients requiring AVR for severe symptomatic AS (16.7%) were found to have a high predicted operative mortality based on logistic EuroSCORE. We have arbitrarily chosen a logistic EuroSCORE > 20, which corresponds to a predicted in-hospital or 30-day mortality > 20%, as our cutoff of high risk based on published reports that have suggested that such patients may be considered inoperable and potentially eligible for alternative catheter-based therapy. Among the patients in our cohort with logistic EuroSCORE > 20, the mean operative mortality predicted by EuroSCORE was 38.7%. However, the actual mortality observed was 11.4%, significantly lower than that predicted by EuroSCORE (P < 0.05). In addition, the EuroSCORE appeared to overestimate operative mortality within all strata of predicted risk. The discrepancy between observed and expected mortality widened as preoperative risk determined by EuroSCORE increased. Although long-term mortality from any cause is significantly higher among patients with EuroSCORE > 20 (log rank P = 0.0001), approximately 60% are alive at five years after operation. Similar findings were recently reported by Brown et al [22]. However, unlike our study, no data are given on longitudinal outcomes.

The EuroSCORE predictive model for cardiac surgery has been extensively studied and found to accurately predict outcomes in a variety of jurisdictions and clinical practices [14-16,30]. The initial additive EuroSCORE model was simple and reproducible and it could readily be used at the patient's bedside for rapid assessment of operative risk. However, what it gained in parsimony, interpretability, and ease of use, it lost in accuracy as it was found to have a tendency to over-predict mortality in low-risk patients and under-predict mortality in high-risk patients [31]. The later-appearing logistic EuroSCORE, utilizing the complete regression equation of the multivariate model to predict mortality risk, was more stable at the extremes of risk and had greater predictive accuracy [26]. However, we have shown that the prognostic performance of the logistic EuroSCORE is poor in patients undergoing AVR, and progressively worsens as risk increases. Similar findings were reported by others in octogenarians undergoing valve operations [21] and in patients undergoing coronary artery bypass graft surgery [32].

It has been shown that subtle inter-institutional differences in the definitions of variables, in the sensitivity of risk factor screening tools, and in the potential presence of various degrees of interdependence and collinearity among covariates assumed to be independent may profoundly affect the performance of a risk prediction model such as the EuroSCORE [33]. However, these influences are likely to be minor in our study given the availability of a nuanced dataset at our institution in which patient information on over 200 variables was prospectively collected at the time of surgery with minimal retrospective chart review, allowing us to adhere to the EuroSCORE definitions of variables as faithfully as possible and to score patients accurately. In no instance was risk factor status imputed secondary to incomplete or missing data fields.

Recent reports suggest that 30–40% of patients in which AVR is indicated are denied surgical treatment, mostly secondary to advanced age and left ventricular dysfunction [34,35]. Less invasive, catheter-based approaches to relieving the obstruction to left ventricular outflow in severe AS were developed in an attempt to target the subset of patients who were not felt to be candidates for conventional open AVR in order to potentially favorably alter their dismal prognosis. Initially, transcatheter AVR was offered to patients on compassionate grounds and was largely reserved for moribund patients with end-stage AS in whom risk factors were felt to be prohibitive for traditional surgery. As experience with transcatheter aortic valve implantation increased with attendant improvements in feasibility, safety, and efficacy profiles, patient selection criteria were expanded and investigators are increasingly relying on EuroSCORE to determine eligibility for catheter-based intervention on the aortic valve. Recent reports evaluating retrograde transfemoral and transapical aortic valve implantation have included patients with mean logistic EuroSCORE 11–35 [6-8,10-13]. In these studies, 30-day mortality rates range from 7 to 22% and the incidence of stroke ranges from 0 to 10%. However, these studies are limited by small numbers of patients and lack long-term data. In our study, the incidence of early stroke was 5.1% in the subgroup of patients with logistic EuroSCORE > 20; 30-day mortality in this high-risk group of patients was 11.4%. Furthermore, five-year survival was acceptable at nearly 60% despite a predicted operative mortality of approximately 40% in this cohort of patients. Taken further, this very same cohort of patients could have been potentially denied life-prolonging operation under current EuroSCORE criteria. However, this study is not a comparison of the effectiveness of conventional AVR and transcatheter aortic valve implantation, nor is it an analysis of the relative merits of one approach over the other. Importantly, our study does not attempt to question the value of important novel approaches to valvular heart disease but questions the validity of using EuroSCORE as a predictive model for high-risk patients with severe AS and suggests that caution should be exercised when using the EuroSCORE risk model to make important management decisions in this complex patient population. This is particularly important when one attempts to make inter-institutional comparisons, compare individual surgeons with different case mixes, predict risk for any single individual patient, or determine patient eligibility for surgery.

### Study limitations

These data reflect the experience of a single center which may limit the study's generalizability. However, our surgical approach to AVR is not likely to be substantially different from the practice at other centers. Also, our overall 30-day mortality of 4.6% following AVR and 11.4% in high-risk patients is within the lower range of post-AVR mortality data reported by the Society of Thoracic Surgeons who have examined more than 40,000 patients [36].

Another important limitation relates to the lack of data on the larger population of patients with severe aortic stenosis who are never presented to our multidisciplinary peer review conference, either because of an a priori judgment made by the health care provider as to a patient's lack of "operability," or a patient's own refusal to be considered for surgery. Therefore, we cannot comment on this wider denominator of untreated patients in the community. However, previous work from our group showed that 88.9% of patients presented to conference eventually undergo operation [23], which is higher than most published reports and is consistent with the existence of a more "aggressive" approach at our institution in accepting patients for surgery. Therefore, it is unlikely that a meaningful selection bias is present in this study such that truly high-risk patients are systematically turned away from surgery, and this limitation does not weaken our data or the study's validity in reporting the outcome of 237 consecutive high-risk patients with severe aortic stenosis and a EuroSCORE of greater than 20. Moreover, in a recent study of patients refused for AVR, mostly by cardiac surgeons, no differences in EuroSCORE were identified between those patients who underwent operation compared to those who did not [37].

## Conclusion

In our consecutive series of patients with severe AS undergoing AVR, we found that the logistic EuroSCORE was not an accurate risk assessment tool in all categories of risk but especially in high-risk patients. Therefore, this predictive model should not be used to determine procedural risk in patients with severe AS. Furthermore, the utilization of the logistic EuroSCORE in the assessment of operability in patients with severe AS may not be appropriate. More accurate risk prediction models are needed for risk-stratifying patients with severe AS.

## Competing interests

The authors declare that they have no competing interests.

## Authors' contributions

DK and JFL conceived the research question, study design and coordination, and drafted the manuscript. DK, DL, and KJB participated in the design of the study and performed the data acquisition and statistical analysis. All authors read and approved the final manuscript.
